# *Staphylococcus aureus* Depends on Eap Proteins for Preventing Degradation of Its Phenol-Soluble Modulin Toxins by Neutrophil Serine Proteases

**DOI:** 10.3389/fimmu.2021.701093

**Published:** 2021-09-06

**Authors:** Dorothee Kretschmer, Ricarda Breitmeyer, Cordula Gekeler, Marco Lebtig, Katja Schlatterer, Mulugeta Nega, Mark Stahl, Daphne Stapels, Suzan Rooijakkers, Andreas Peschel

**Affiliations:** ^1^Interfaculty Institute for Microbiology and Infection Medicine Tübingen (IMIT), Infection Biology, University of Tübingen, Tübingen, Germany; ^2^German Center for Infection Research, Partner Site Tübingen, Tübingen, Germany; ^3^Cluster of Excellence EXC2124 “Controlling Microbes to Fight Infections”, Tübingen, Germany; ^4^Interfaculty Institute for Microbiology and Infection Medicine Tübingen (IMIT), Microbial Genetics, University of Tübingen, Tübingen, Germany; ^5^Center for Plant Molecular Biology (ZMBP), University of Tübingen, Tübingen, Germany; ^6^Department of Medical Microbiology, University Medical Center Utrecht, Utrecht University, Utrecht, Netherlands

**Keywords:** *Staphylococci*, *Staphylococcus aureus*, neutrophil serine proteases, neutrophil serine protease inhibitors, phenol-soluble modulins, formyl-peptide receptor 2

## Abstract

Neutrophil granulocytes act as a first line of defense against pathogenic staphylococci. However, *Staphylococcus aureus* has a remarkable capacity to survive neutrophil killing, which distinguishes it from the less-pathogenic *Staphylococcus epidermidis.* Both species release phenol-soluble modulin (PSM) toxins, which activate the neutrophil formyl-peptide receptor 2 (FPR2) to promote neutrophil influx and phagocytosis, and which disrupt neutrophils or their phagosomal membranes at high concentrations. We show here that the neutrophil serine proteases (NSPs) neutrophil elastase, cathepsin G and proteinase 3, which are released into the extracellular space or the phagosome upon neutrophil FPR2 stimulation, effectively degrade PSMs thereby preventing their capacity to activate and destroy neutrophils. Notably, *S. aureus*, but not *S. epidermidis*, secretes potent NSP-inhibitory proteins, Eap, EapH1, EapH2, which prevented the degradation of PSMs by NSPs. Accordingly, a *S. aureus* mutant lacking all three NSP inhibitory proteins was less effective in activating and destroying neutrophils and it survived less well in the presence of neutrophils than the parental strain. We show that Eap proteins promote pathology *via* PSM-mediated FPR2 activation since murine intraperitoneal infection with the *S. aureus* parental but not with the NSP inhibitors mutant strain, led to a significantly higher bacterial load in the peritoneum and kidneys of mFpr2^-/-^ compared to wild-type mice. These data demonstrate that NSPs can very effectively detoxify some of the most potent staphylococcal toxins and that the prominent human pathogen *S. aureus* has developed efficient inhibitors to preserve PSM functions. Preventing PSM degradation during infection represents an important survival strategy to ensure FPR2 activation.

## Introduction

Neutrophils are among the most important leukocytes in the host defense against the opportunistic bacterial pathogen *Staphylococcus aureus* (1). After engulfing and sequestering *S. aureus* into the phagocytic vacuole, neutrophils deploy a variety of oxidative and non-oxidative weapons to kill the bacteria (2). While only those mechanisms that generate oxidants are considered to play an essential role in killing *S. aureus* within the phagocytic vacuole (3, 4), the non-oxidative ones may be essential for the killing of other types of bacteria (5–8). Among the non-oxidative antimicrobial weapons are the neutrophil serine proteases (NSPs), which are stored - tightly bound to proteoglycans - within intracellular acidic granules (9). Upon phagocytosis of microorganisms, acidic granules fuse with and discharge their content into the phagolysosome, where NSPs get activated and presumably participate to the degradation of bacteria (10). Alternatively, in response to neutrophil activation by specific stimuli, acidic granules discharge their content, including NSPs, to the extracellular space (11). Different NSPs with a role in bacterial infection have been identified: neutrophil elastase (NE), cathepsin G (CG), proteinase 3 (PR3) and the less abundant NSP4 (12, 13).

It has been shown that NSPs have distinct effects on different kinds of bacteria. NE can directly kill Gram-negative *Escherichia coli* and *Klebsiella pneumoniae* (7, 14). In the case of *E. coli*, this activity depends on the cleavage of its outer membrane protein OmpA. Furthermore, the Gram-positive *Streptococcus pneumoniae* is known to be killed by the concerted action of the NE, CG, and PR3 within the phagocytic vacuole (5, 6). Recent data show that NSPs, especially CG, can degrade biofilms of *S. aureus* (15) but cannot directly kill *S. aureus*. This observation is probably due to the ability of CG, to cleave of the active domain of the adhesion protein clumping factor A (ClfA) (16). In addition, neutrophil extracellular traps associated α-toxin can probably also be degraded by NSP (17). Nonetheless, mice that lack NE and CG are more susceptible to streptococcal infections, but are as susceptible as wild-type mice to *S. aureus* infection. *S. aureus* is known to secrete a set of three different NSP inhibitors, the extracellular adherence protein Eap, and the orphan Eap homologs EapH1 and EapH2. The importance of these NSP inhibitors for *S. aureus* is reflected in the fact that all *S. aureus* strains encode at least two members of the Eap family genes (18). Eap is among the secreted and cell-bound virulence factors induced by the Sae two-component system upon phagocytosis. Therefore, *eap* is expressed in the phagosome during *in vivo* and *in vitro* infection (19). Little is known about the regulation of *eapH1* and *eapH2* expression, apart from the fact that they are expressed during *in vivo* infection, possibly in a differential manner (18). *S. aureus* immune evasion proteins CHIPS and FLIPr are vulnerable to proteolytic inactivation by NSPs while others with immune-escape functions, e.g. SCIN A, SCIN B and SCIN C, have differential cleavage sensitivity towards NSPs, with SCIN A being resistant to cleavage (13). Besides these immune evasion proteins, staphylococci secrete high amounts of peptide toxins, the phenol-soluble modulins (PSMs), the expression of which is regulated by the two-component system Agr (20–22). In contrast to other Agr regulated virulence factors, which are under the control of the regulatory RNAIII, all PSMs but the δ-toxin are directly regulated by the response regulator AgrA. Whether PSMs or other *S. aureus* toxins are also susceptible to cleavage by NSPs has remained unclear. PSMs activate the formyl-peptide receptor 2 (FPR2) on the surface of neutrophils, thereby inducing chemotaxis, and promoting phagocytosis and oxidative burst by neutrophils. Notably, PSMs are important for the recruitment, activation and degranulation of Neutrophils during early *S. aureus* intraperitoneal mouse infection in an FPR2 dependent manner (23–25). At higher concentrations, especially alpha PSMs, lyse leukocytes in an FPR2 independent manner. Lipoproteins such as high-, low- and very low-density lipoproteins in human serum are able to inactivate secreted PSMs (26). However, PSMs are also secreted in serum-free environments, for instance when bacteria reside in the phagosome of neutrophils (27), and facilitate bacterial escape from the phagolysosome into the cytosol (28, 29). In the leukocyte cytoplasm, *S. aureus* is protected from the weapons of the granules. We hypothesized that NSPs degrade PSMs and that *S. aureus*, in contrast to other PSM-producing staphylococci, may prevent the breakdown and maintain the activity of its PSMs by secreting the NSPs inhibitors Eap, EapH1 and EapH2.

Our data demonstrate that PSMs of staphylococci induce degranulation of neutrophils. In turn, degranulated NSPs can degrade PSMs. Thus, culture filtrates of an *S. aureus* mutant lacking the serine protease inhibitors Eap, EapH1 and EapH2 enhance release of NE by neutrophils compared to the corresponding wild-type, leading again to increased PSM degradation. Consequently, killing of the triple NSP inhibitor mutant as well as *S. epidermidis* by neutrophils is significantly enhanced compared to wild-type *S. aureus*. High Performance Liquid Chromatography-Mass Spectrometry (HPLC-MS) data confirmed that neutrophils degraded PSMs in culture filtrates of the NSP inhibitor mutant more efficiently than PSMs of the wild-type. These data suggest that the maintenance of PSM activity in the presence of neutrophils is a crucial difference between *S. aureus* and coagulase-negative staphylococci.

## Material and Methods

### Isolation of Human Neutrophils

Human neutrophils were isolated from healthy blood donors by density gradient centrifugation as previously described (23).

### Peptides and Enzymes

Formylated PSMα3, PSMα2 and δ-toxin were kindly provided by Stefan Stevanovic (Dep. of Immunology, Tuebingen). Purchased were: human neutrophil elastase (PanReac AppliChem), human neutrophil cathepsin G (MyBioSource), human neutrophil proteinase 3 (MyBioSource), and Phenylmethylsulfonyl fluoride (PMSF, Roche). Recombinant EAP (2mg/ml), EAPH1 (2mg/ml), EAPH2 (1,5mg/ml), were kindly provided by S. Rooijakkers (University Medical Center Utrecht, The Netherlands). Formylated S. *epidermidis* PSMα, PSMϵ and PSMδ were synthesized by EMC Microcollections, Tuebingen.

### Bacteria

Bacteria used in this study were *S. epidermidis* 1457, *S. epidermidis* 1457*Δagr* (30), *Staphylococcus aureus* USA300 LAC (20), USA300*Δαβδ* (31). Mutants generated in this study USA300 LAC *Δeap*, USA300 LAC *ΔeapH1*, USA300 LAC *ΔeapH2::ermB* and USA300 LAC *ΔeapΔeapH1eEapH2::ermB*. Bacterial strains were grown at 37°C in tryptic soy broth (TSB) for 17 h under agitation (160 rpm). Bacterial culture filtrates were obtained by centrifugation (10 min, 4°C and 5000 x g) of overnight cultures grown in tryptic soy broth (TSB) and filtered through 0.22-μm pore size filters (Merck Millipore). Staphylococcal cultures were standardized to the same optical density.

### Construction of Mutant Strains

For deletion of *eap* or *eapH1* in USA300 LAC, the knockout plasmid pKOR1-Eap or pKOR1-EapH1 containing the upstream and the downstream flanking regions of *eap* or *eapH1*, kindly provided by S. Rooijakkers, was used. Deletion was performed as described (18). For deletion of *eapH1 in* USA300*Δeap*, the knockout plasmid pBASE6 containing the upstream and the downstream flanking regions of *eapH1* was constructed. The flanking regions were amplified by PCR from the genome of USA300 LAC, the upstream one using primers F1_up (EcoRI)(CAACGAATTCTTTAACATGCAGTGTTATCCC) and F1_down (BglII) (GATATTACACTAGATCTATAACACGTTTC) and the downstream one using primers F2_up (BglII)) (TGAAAATAGATCTATAGGGCAAGCGCTGAA) and F2_down (SalI) (GGTATCGGTCGACTAACAGGTTCAAACGG). The amplified fragments were cut with BglII. These fragments were then ligated with vector pBASE6 digested by EcoRI and SalI resulting in pBASE6ΔeapH1, which was transformed into *Escherichia coli* DC10b. The clones containing plasmid pBASE6ΔeapH1 were isolated, purified, and sequenced. The correct plasmid pBASE6 ΔeapH1 was subsequently transformed by electroporation into *S. aureus* RN4220 as an intermediary host and then transformed into *S. aureus* USA300 LAC Δ*eap*. The procedure for deletion of EapH1 from USA300 LAC Δ*eap* was followed as described previously. The obtained *eapH1* gene replacement mutant strains were verified by PCR.

The triple mutant and single mutant of EapH2 was obtained by transducing the *eapH2::ermB* mutation into the *S. aureus* strain USA300*ΔeapΔH1* or wild-type USA300 LAC using W11 lysates of strains NARSA strain ID: NE111. Transductions were verified by PCR and Sequencing (GATC). For this, the NARSA strain NE111 was cultivated overnight in TSB plus erythromycin (2.5 µg/ml). 20 ml of Phage Φ11 (>1x10^9^ p.f.u./ml) was prepared and CaCl_2_ was added (5 mM). Bacteria were added to a final OD_600_ of 1.0. For phage adsorption, the mixture was incubated for 45 minutes at 37°C and for 5 hours at 40 rpm, 30°C. After centrifugation the suspension was sterile filtered (0.22 μm pore filter). The corresponding recipient target strain USA300*ΔeapΔeapH1* was incubated overnight at 37°C in TSB and bacteria were diluted to a final OD_600_ of 0.5. Different ratios of recipient culture to phage lysate were used during transduction. Therefore, 200 μl, 150 μl, 100 μl or 20 μl of the recipient strain culture were spin down, resuspended in 200 μl phage buffer and different amounts of phage lysate was added. After incubation (15 min, 350 rpm, 37°C) 200µl of each mixture were streaked on TSB plates containing erythromycin and incubated for 48 hours. Single colonies were grown over night in TSB containing erythromycin to isolate genomic DNA (Nucleospin Tissue Kit). gDNAs were used as template for PCR targeting EapH2 using primers 0883_5’_fw and 0883_3’_rev to amplify the whole EapH2 locus (**Supplemental Table1**). Mutations were confirmed by gel electrophoresis and sequencing (GATC Biotech).

### Degranulation Assay

4x10^5^ neutrophils/well in HBSS+0.2 mM BSA +1mM HEPES were incubated with 100 μl Cytochalasin B (30µM) in 96 well plates for 15 minutes on ice, followed by 15 minutes of incubation at 37°C and 5% CO_2_. After centrifugation, supernatants were removed and the pellets were stimulated for 10 minutes (37°C, 5% CO_2_) with 100 μl of diluted bacterial culture supernatants (0.75% and 0.36%), fMLF or PSMs to induce degranulation. The stimulation was stopped *via* centrifugation for 10 minutes at 250 x g and 4°C. 20 μl of the supernatants were then transferred into a black 96 well flat bottom plate and 20 μl of a 10 mM 4-Methylumbelliferyl β-d-glucuronide hydrate stock solution in 0.1 M Sodium Acetate (pH 4.0) were added and mixed briefly. After 15 minutes at 37°C and 5% CO_2_ the reaction was stopped by adding 250 μl of stop solution (50 mM glycine, 5 mM EDTA in ddH_2_O). Degranulation results in the release of β-glucuronidase, and its activity can be measured in a microplate reader by monitoring cleavage of the substrate 4-Methylumbelliferyl β-d-glucuronide (Sigma), which releases the fluorescent moiety 4-Methylumbelliferyl (excitation wavelength of 365 nm and an emission wavelength of 460 nm).

### Elastase Activity Assay

NE can be quantified by monitoring cleavage of the elastase substrate (MeOSuc-AAPV-AMC; Calbiochem). 5x10^5^ neutrophils per well were seeded into wells of a 96 well round-bottom plate and PSMs of either *S. aureus* (PSMα2, α3 or δ-toxin) or *S. epidermidis* (PSMα, δ or ε) or culture filtrates of *S. epidermidis* (3%) were added. To analyze inhibition of NE activity, recombinant Eap, EapH1 or EapH2 was added (each 5µg/ml). Neutrophils and PSMs or bacterial culture filtrates were incubated for one hour at 37°C and 5% CO_2_. Subsequently, the plate was centrifuged (5 min, 300 x *g*, RT) and 100 μl of the supernatant were transferred into a new flat bottom 96 well plate. An elastase standard curve was created by two-fold dilution of an NE stock solution from 5 μg/ml to 78.125 ng/ml. TSB (3%) alone served as negative control. 100 μl of 2 mM elastase substrate was added and the mixture was incubated for 30 minutes (37°C, 5% CO_2_). Absorbance (405nm) as readout of NE activity was measured in a microplate reader.

### Inactivation of Synthetic PSMs by NSPs

The three different human serine proteases human NE, PR3, 0.01 U and CG, 0.01 U were investigated and their potential to inactivate PSMα3 was compared. As NE was the most active protease, usually NE was used in the described experiments. 28 ng/ml NE (stock: 1.4 mg/ml) were incubated for one hour at 37°C in RPMI plus 0.05% HSA to inactivate 500 nM PSMα3 in a total volume of 120 μl (0.012 μl NE in 60 μl RPMI). To avoid further degradation of PSMα3, all stimulations were prepared freshly and analyzed in different assays on the same day.

### Inactivation of PSMs in Bacterial Culture Filtrates by Neutrophils

Culture filtrates of different bacterial strains (USA300, *USA300ΔeapΔeapHIΔeapHII::ermB*, *S. epidermidis* RN1457) were incubated with different amounts of neutrophils to investigate the potential of neutrophils to inactivate their PSM activity. Therefore, 100µl of sterile filtered overnight culture were incubated with 100µl neutrophils in RPMI at the indicated concentrations for six or 24 hours as described in the figure legends. After centrifugation, supernatants were collected and frozen at -20° until they were further analyzed for their FPR2 activity, for truncated PSM fragments and their cytotoxicity.

### Calcium Mobilization in HL60 FPR2 Cells

HL60 cells stably transfected with human FPR2/ALX (HL60-FPR2) have been described recently (23). These cells were grown in RPMI medium (Biochrom) supplemented with 10% FCS (Sigma-Aldrich), 20 mM Hepes (Biochrom), penicillin (100 units/ml), streptomycin (100 µg/ml) (Gibco), 1 × Glutamax (Gibco) and in the presence of G418 (Biochrom) at a final concentration of 1 mg/ml. Neutrophils or HL60-FPR2 cells were stimulated with different bacterial culture filtrates (USA300, USA300*Δαβδ*, USA300*ΔeapΔH1ΔH2::ermB*, *S. epidermidis* RN1457) or FPR2 ligands (PSMα3, PSMε) as described in the figure legends. Calcium fluxes were analyzed by stimulating cells loaded with Fluo-3-AM (Molecular Probes) and monitoring fluorescence with a FACSCalibur or a FACS Fortessa flow cytometer (BD Biosciences) as described recently (23).

### Cytotoxicity Assay

100 μl of synthetic PSMs, protease degraded PSMs or filtered bacterial culture supernatants were added to wells of a 96-well round bottom culture plate containing 10^5^ neutrophils in 100 μl and plates were incubated at 37°C for up to 3 h. At the desired times, the stimulation was stopped by centrifugation for 10 minutes at 300 x g at RT, and cell lysis was determined by measuring the release of lactate dehydrogenase (LDH) using the Cytotoxicity Detection Kit (Roche Applied Sciences), according to the manufacturer’s instructions. As control, 100 μl TSB was diluted in RPMI without phenol red, mixed with human neutrophils (10^6^) and tested for its ability to cause cell lysis. As control, lysis of HL60 cells was tested in pure RPMI/TSB without phenol red.

## CD11b Expression

Sterile filtered culture filtrates of *S. aureus* USA300 (0,36% in RPMI) or TSB were incubated for 24 hours either with or without addition of the NSP inhibitor PMSF and neutrophils. Activation of human neutrophils was determined by measuring surface expression of CD11b. Neutrophils were incubated with the treated culture filtrates at 37°C with rotation for 60 min as described elsewhere (24). Cells were stained with a PE-labeled antibody against CD11b (mAb 44; BD Biosciences) or isotype control antibody (BD Biosciences) as described previously. Then, neutrophils were analyzed on a FACS Fortessa cytometer (BD Biosciences)

### Oxidative Burst

10^5^ neutrophils in Hank Balanced Salt Solution (HBSS/0.05% HSA) were stimulated with 100 μl of digested (with NE, CG or PR3 as described before) or nondigested PSMα3 together with 100 μl Luminol-solution in a 96 well, black, flat bottom plate. ROS production by human neutrophils was measured over a time period of one hour by monitoring luminol-amplified chemiluminescence using 282 µM luminol (Sigma Aldrich). Chemiluminescence was determined in a luminescence microplate reader (BMG Labtech).

### Western Blot

PSMα3 (1,3µg) was digested either with 0,15 unit’s NE, CG or PR3 for one hour at 37°C with or without addition of PMSF (100µM) or 2µg Eap, 4µgEapH1 or 4µg EapH2. For digestion of PSMα3 by neutrophils, 25µl PSMα3 (20µM) was incubated for one hour with or without 5x10^6^ neutrophils in 100µl RPMI at 37°C, 5% CO_2_. After centrifugation 10µl of the supernatant was used for western Blot analysis. After denaturation of the samples for two minutes at 85°C and addition of Tricine Sample Buffer, samples were subjected to a 10-20%- Tricine SDS gradient gel (Thermo Fischer Scientific). Then, proteins were blotted to a nitrocellulose membrane and blocked overnight using blocking buffer. After washing PSM α3 was visualized using rabbit anti-PSMα3 serum (kindly provided by Michael Otto) at a concentration of 1:10000 (for one hour, 4°C, under agitation). After washing with wash buffer (Tris buffered saline, BioRad +0,05% Tween, Merck), the secondary antibody goat anti rabbit IgG (Dylight 800 conjugate (LI-COR #2557) was added (diluted 1:10000 in blocking buffer) and incubated for one hour at 4°C. Protein bands were detected with the Licor Odyssey CLx.

### Killing Assay

*S. aureus* strains USA300 LAC or USA300*ΔeapΔH1ΔH2::ermB* were grown overnight at 37°C and 160 rpm in TSB medium. Next, the bacteria were washed three times with PBS and opsonized with 10% human pooled serum (Hospital Tübingen) in RPMI for one hour at 37°C. 5x10^5^ neutrophils in 200 μl were incubated with 50µl bacteria in the desired MOI. Stimulation was done for up to 4h at 37°C and 5% CO_2_. Then neutrophils were lysed with ddH_2_O for 15 min at 4°C, 1000 rpm. Serial dilutions of the samples were plated on TSA plates using an IUL EDDY Jet 2 spiral plater. Plates were incubated overnight at 37°C and counted the next day using an IUL Flash & Go instrument.

### HPLC Analysis of PSMs in Culture Filtrates and Determination of Truncated PSMs *via* LC/MS

Samples were prepared as described before for inactivation of PSMs in bacterial culture filtrates by neutrophils. The corresponding supernatants were concentrated 3x using a speedvac vacuum concentrator. PSM peptides were separated from the supernatant by reversed-phase chromatography using an XBridge C8 5µm, 4.6 x 150 mm column (Waters). A linear gradient from 0.1% TFA (buffer A) in water to acetonitrile containing 0.08% TFA (buffer B) for 15 minutes with additional 5 minutes of 100% buffer B at a flow rate of 1ml/min was used and a 50 µl sample volume was injected. Peaks were detected at 210 nm. Enzymatic peptide cleavages were revealed by LC/MS analysis. Therefore, 5 µl of the different concentrated samples were analyzed with an Acquity HPLC – SynaptG2 LC/MS system (Waters, Manchester, England). Chromatography was done on a Waters Acquity C18 HSST3 2,1x100 mm, 1,8 µm column operated at a flow rate of 200 µl/min and 35°C. Separation was done with a 25 min water/methanol gradient (both solvents contained 0,1% formic acid). The mass spectrometer was operated in positive electrospray ionization mode with an ionization voltage of 3000 V, a scan range from 50 to 2000 m/z, a scan time of 0.5 sec and a resolution of 10000. MS and MSE traces were recorded in parallel. Peptides and their fragments were identified based on their exact mass, charge state and partly all ion fragmentation patterns. For quantification extract ion chromatograms were generated with a mass window of 0.02 Da and integrated (**Supplemental Table 1**).

### Mouse Infection Assay

All experimental procedures involving mice were carried out according to protocols approved by the Animal Ethics Committees of the Regierungspräsidium Tübingen (IMIT1/18). We used for the animal experiment’s female C57BL/6J mFpr2^-/-^ with homozygous chromosomal deletion of both fpr2 alleles. mFpr2^-/-^ mice have been previously described (32). Mice were held under specific pathogen-free conditions, provided food and water ad libitum. C57BL/6J mice (Envigo, Netherlands), were used as wild-type control mice. For the mouse peritonitis model, six- to eight- weeks-old female C57BL/6J wild-type and mFpr2^-/-^ mice were used. 5 x 10^8^ CFUs of either *S. aureus* USA300 LAC or the USA300 *ΔeapΔH1ΔH2::ermB* were injected in the peritoneum. At six hours after infection mice were euthanized with CO_2_ and peritoneal exudates were collected and surface receptor expression of peripheral blood leukocytes was determined. For this purpose, erythrocytes were lysed and leukocytes were stained with monoclonal antibodies specific for or CD11b, Ly6G and F480 (all Miltenyi). Ly6G was used as neutrophil marker. The staining was analysed by a FACS LSR Fortessa X-20 (BD). The numbers of immigrated cells were detected by counting in the peritoneal exudates. Subsequently, liver, kidney and spleen were homogenized and colony forming units (CFUs) in organs were determined by plating serial dilutions on agar plates.

### Ethics Statement

The institutional review board (IRB) of the University of Tübingen approved the study and all adult subjects donating blood provided informed consent. This study was done in accordance with the ethics committee of the medical faculty of the University of Tübingen that approved the study, Approval number 015/2014 BO2.

### Statistics

Statistical analysis was performed using Graph Pad Prism 8.0. (GraphPad Software, La Jolla, USA). Unpaired two-tailed Student’s t test or Mann-Whitney-U test was used to compare two data groups and one-way ANOVA with Dunnett’s multiple comparisons test was used to compare more groups, unless otherwise noted.

## Results

### PSMs Induce Degranulation and the Release of NE

**Figure 1:**
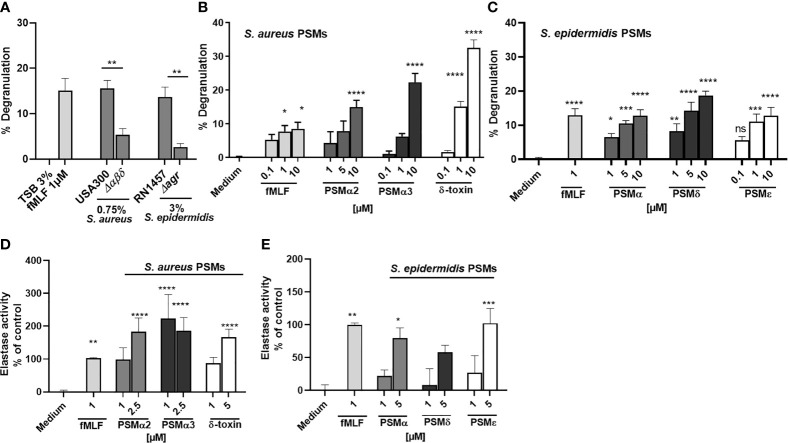
PSMs in culture filtrates of *S. aureus* and *S. epidermidis* induce neutrophil degranulation and NE release. Neutrophils (polymorphonuclear leukocytes=PMNs) were stimulated with **(A)** culture filtrates of *S. aureus* (0,75%), *S. epidermidis* (3%) or the indicated isogenic PSM or *agr* mutant **(B)** with fMLF, PSMα2, PSMα3 or δ-toxin or **(C)** with *S. epidermidis* PSMα, PSMδ or PSMε, and degranulation (β-glucuronidase release) was analyzed. **(D)** Neutrophils were stimulated with the indicated *S. aureus* or **(E)** *S. epidermidis* PSMs and elastase activity was analyzed. Data represents mean and SEM of at least three independent experiments. ns, not significant, *P < 0.05; **P < 0.01; ***P < 0.001; ****P < 0.0001 significant difference versus the indicated wild-type strains as calculated by paired two-tailed Student’s t tests **(A)** or one-way ANOVA with Dunnett’s multiple comparisons test **(B–E)**.

Since it has been shown that FPR ligands induce the release of granules by neutrophils, and PSMs are known to activate neutrophils *via* FPR2 (23, 24), we wondered whether PSM stimulation of FPR2 induces degranulation of neutrophils. Stimulation of neutrophils with culture filtrates of PSM-releasing *S. aureus* USA300 and *S. epidermidis* RN1457 induced the release of β-glucuronidase, a readout for degranulation (33), whereas culture filtrates of a PSM-deficient mutant of USA300 (*Δαβδ*) or a PSM non-producing *agr* mutant of RN1457(*Δagr*) induce significant less degranulation (**Figure 1A**). When we used synthetic formylated PSMα2, PSMα3 or δ-toxin of *S. aureus* (**Figure 1B**) as well as formylated PSMα, PSMδ or PSMϵ of *S. epidermidis* (**Figure 1C**) to stimulate neutrophils, we observed that all tested PSMs of *S. aureus* and *S. epidermidis* induced degranulation and neutrophil elastase release as quantified by measuring the activity of released elastase (**Figures 1D, E**). These data indicated that PSMs induced neutrophil degranulation and elastase release, but whether and how neutrophils may influence PSM activity remained unclear.

### Neutrophils Inhibit the PSM-Mediated Activation of FPR2 Through the Activity of NSPs

**Figure 2:**
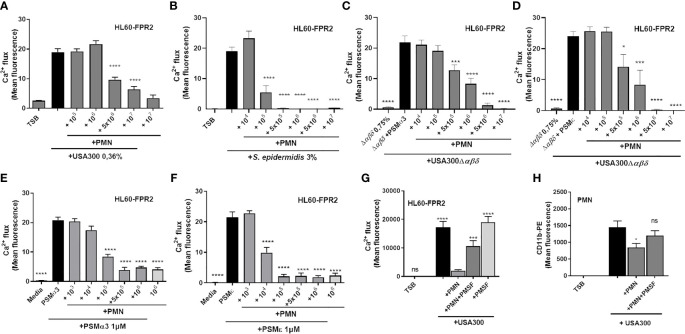
Neutrophils inactivate PSM-mediated FPR2 activity of culture filtrates by NSPs. Culture filtrates of *S. aureus* USA300 **(A)** or *S. epidermidis* RN1457 **(B)** were incubated for six hours with increasing amounts of neutrophils. FPR2 transfected HL60 were stimulated with these treated culture filtrates and FPR2 activity was analyzed. PSMα3 **(C)** or PSMε **(D)** were incubated with neutrophils. FPR2 transfected HL60 were stimulated with these supernatants and PSM activity was analyzed. Addition of PSMα3 **(E)** or PSMε **(F)** to culture filtrates of a *S. aureus* PSM mutant induces FPR2 activation, an effect that can be dose-dependently inactivated by increasing amounts of neutrophils. Preincubation of neutrophils with the NSP inhibitor PMSF prevents inactivation of FPR2 activity **(G)** or CD11b upregulation by culture filtrates **(H)**. Data represents mean and SEM of at least three independent experiments. ns, not significant, *P < 0.05; ***P < 0.001; ****P < 0.0001 significant difference versus the indicated untreated control **(A–F, H)** or the neutrophil-treated control **(G)** as calculated by one-way ANOVA with Dunnett’s multiple comparisons test **(A–H)**.

To address whether neutrophils may influence PSM activity, we analyzed the capacity of neutrophils to inhibit the activation of FPR2 by culture filtrates of PSM producer strains. Therefore, we incubated culture filtrates of *S. aureus* USA300, USA300Δαβδ or *S. epidermidis* RN1457 with or without addition of neutrophils for six hours to investigate the potential of neutrophils to inactivate their PSM activity. After removal of neutrophils by centrifugation, we stimulated human leukemia cells 60 overexpressing FPR2 (HL60-FPR2) with these culture filtrates and observed that pretreatment with neutrophils prevented FPR2 activation in a dose-dependent manner (**Figure 2A**). We previously verified that non-transfected HL60 cells lacking FPR2, are not activated after stimulation with culture filtrates at the indicated concentrations (24). Interestingly, the treatment of *S. epidermidis* culture filtrates with neutrophils inhibited FPR2 activation in HL60-FPR2 cells with even higher efficiency (**Figure 2B**). To demonstrate that indeed the PSMs in the culture filtrates were responsible for the observed FPR2 activity, we supplemented culture filtrates of an *S. aureus* PSM mutant strain with similar amounts of either synthetic PSMα3 (**Figure 2C**) or PSMϵ (**Figure 2D**) and incubated them with increasing numbers of neutrophils. We observed in both cases comparable inactivation of FPR2 activity. To check whether also incubation of synthetic PSMs with neutrophils prevented FPR2 activation, we stimulated HL60-FPR2 cells exclusively with synthetic PSMα3 and PSMϵ (**Figures 2E, F**) in the presence of neutrophils. We found that treatment of PSMα3 as well as PSMϵ with increasing numbers of neutrophils led to a dose-dependent loss of FPR2 activation. Since PSMs induced degranulation and release of NE, we assessed whether the released NSPs were responsible for the inactivation of PSMs. Treatment of HL60-FPR2 cells with culture filtrates of a PSM producing *S. aureus* strain in the simultaneous presence of neutrophils and of the synthetic NSP inhibitor phenylmethylsulfonyl fluoride (PMSF) rescued PSM-mediated FPR2 activation (**Figure 2G**). Similarly, upregulation of CD11b, a marker of neutrophil activation, was observed when neutrophils were incubated with culture filtrates of a PSM-producing *S. aureus* strain in the presence of PMSF but not with PSM-deficient culture filtrates (**Figure 2H**).

### NSPs Degrade PSMα3 Thereby Preventing FPR2 Activation

**Figure 3:**
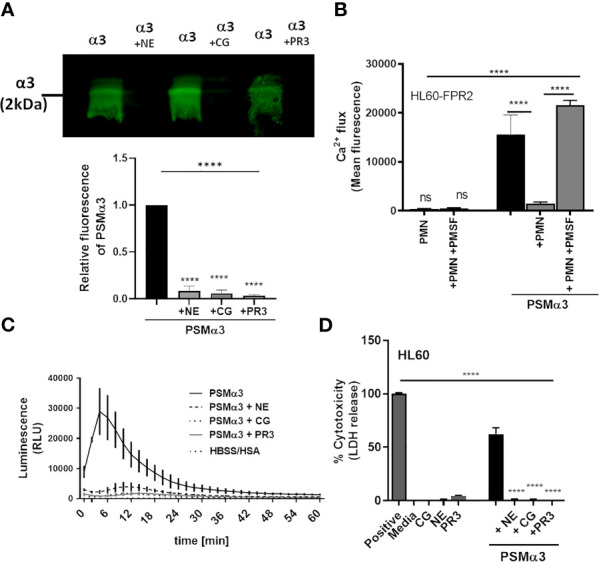
NSPs degrade PSMα3. Addition of NSPs NE, CG or PR3 results in degradation of PSMα3 **(A)**. Incubation of neutrophils with the NSP inhibitor PMSF prevents inhibition of PSMα3-mediated FPR2 activity **(B)**. Inactivation of PSMα3 by NE, CG or PR3 prevents oxidative burst of neutrophils **(C)** and leads to loss of cytotoxicity **(D)**. Data represent mean +/-SEM of three independent experiments **(A–D)**. ns, not significant; ****P < 0.0001 significant difference versus untreated control **(A)**, the neutrophil-treated control **(B)** or the PSMα3-treated control **(D)** as calculated by one-way ANOVA with Dunnett’s multiple comparisons test **(A–D)**.

**Figure 4:**
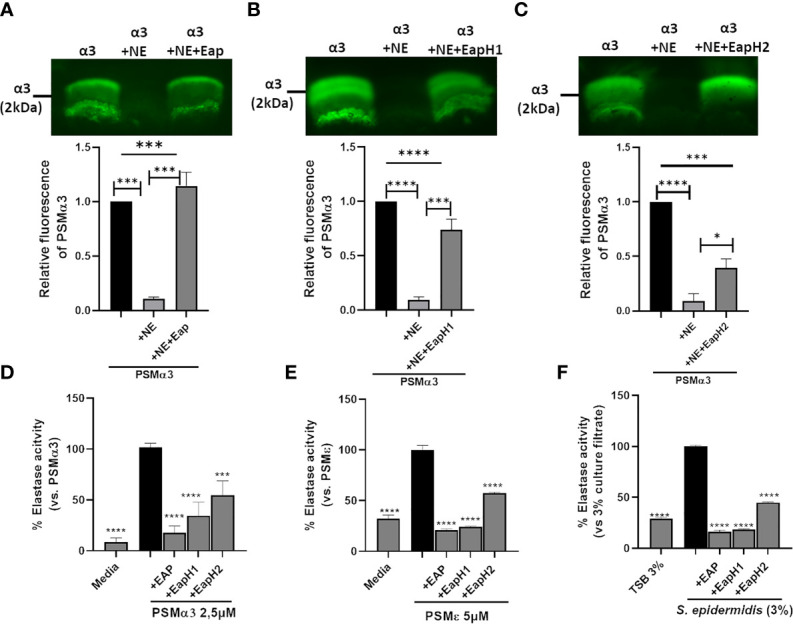
*S. aureus*-derived NSP inhibitors prevent degradation of PSMα3. Western blot of PSMα3 after incubation with NE alone (0.15 U) or with 2 µg/ml Eap **(A)**, EapH1 (4 µg/ml) **(B)** or EapH2 (4 µg/ml) **(C)**. NE activity of neutrophils +/-preincubation with Eap, EapH1 or EapH2 after stimulation with PSMα3 **(D)**, PSMε **(E)**. NE activity of neutrophils incubated with *S. epidermidis* culture filtrates (3%) +/- recombinant Eap, EapH1 or EapH2 (each 5µg/ml). Data represent one experiment out of three **(A–C)** and mean +/-SEM of three independent experiments **(A–D)**. *P < 0.05; ***P < 0.001; ****P < 0.0001 significant difference *versus* NE-treated PSMα3 **(A–C)**, the PSMα3- **(D)** or PSMε-treated control **(E)** as well as the culture filtrate treated control **(F)** as calculated by one-way ANOVA with Dunnett’s multiple comparisons test.

We confirmed by western blotting that upon incubation with NE, CG or PR3, PSMα3 was readily degraded (**Figure 3A**). In support of this finding, pre-incubation of neutrophils with the NSP inhibitor PMSF averted degradation of PSMα3 (**Supplemental Figure 1A**) and maintained the activation of FPR2 in HL60-FPR2 cells (**Figure 3B**). Treatment with NE, CG and PR3 also abolished the ability of PSMα3 to induce oxidative burst in neutrophils and cytotoxicity toward HL60 cells (**Figures 3C, D**). We noticed that relatively high numbers of neutrophils were required to inactivate PSMs in highly diluted culture filtrates (0.36%) of *S. aureus* US300 compared to inactivation of 1 µM synthetic PSMα3. Therefore, we hypothesized that the NSP inhibitors of *S. aureus* Eap, EapH1 and EapH2, could prevent the degradation of PSMα3 by NSPs. Indeed, we observed that all three NSP inhibitors of *S. aureus* prevented degradation of PSMα3 (**Figures 4A–C**) and inhibited the activity of the released NE (**Figure 4D**). Furthermore, all three NSP inhibitors of *S. aureus* also inhibited PSMε-induced NE activity (**Figure 4E**). Addition of recombinant NSP inhibitors Eap, EapH1 or EapH2 to *S. epidermidis* culture filtrates inhibited the activity of the NE released from neutrophils as well (**Figure 4F**). These finding demonstrate that human NSPs can neutralize some of the most potent staphylococcal toxins and that *S. aureus* protects its PSMs from degradation with the help of its NSP inhibitors.

### Lack of NSP Inhibitors in *S. aureus* Enhances Degradation of PSMs by Neutrophils in Culture Filtrates

**Figure 5:**
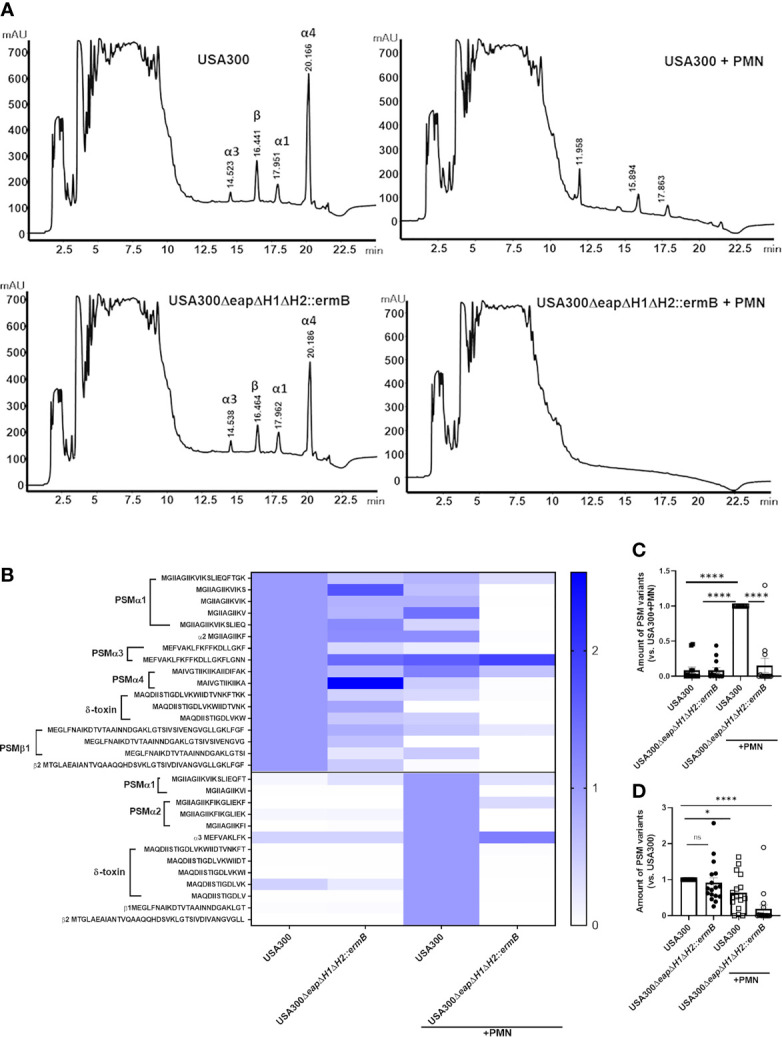
NSPs enhance degradation of PSMs by neutrophils in culture filtrates of USA300. No pre-incubation or prior incubation of culture filtrates of wild-type USA300 with neutrophils for 24 h leads to disappearance of original peaks representative for α and β-PSMs in the HPLC chromatogram, but also to the appearance of new peaks. Treatment of culture filtrates of USA300*ΔeapΔH1ΔH2::ermB* with neutrophils only leads to disappearance of nearly all peaks representative for PSMs **(A)**. Heat map of HPLC/MS Analysis of neutrophil-treated or untreated culture filtrates show that incubation of USA300 indeed leads to disappearance of a few PSM fragments, found in untreated culture filtrates, but also to the appearance of new fragments. In neutrophil-treated culture filtrates of the USA300*ΔeapΔH1ΔH2::ermB* mutant most truncated fragments are absent. Heat map represents data normalized towards USA300 (above the line) or towards USA300 plus neutrophils (below the line) **(B)**. C-terminally truncated PSM fragments with highest abundance in neutrophil-treated culture filtrates of USA300 **(C)** or PSM fragments with highest abundance in untreated culture filtrates of USA300 **(D)**. Data represent mean and SEM of three different culture filtrates of USA300 or USA300*ΔeapΔH1ΔH2::ermB*, each incubated with or without neutrophils of two different donors. ns, not significant, *P < 0.05; ****P < 0.0001 significant difference *versus* USA300+neutrophils **(C)** or USA300 **(D)** as calculated by one-way ANOVA with Dunnett’s multiple comparisons test.

To analyze the consequence of absence of NSP inhibitors in *S. aureus* we generated three single (USA300*Δeap*, USA300*ΔeapH1*, USA300*ΔeapH2::ermB*) and a triple mutant of USA300 (USA300*ΔeapΔH1ΔH2::ermB*), the latter lacking all three NSP inhibitors. USA300 and the triple NSP inhibitor mutant produced the same amounts of PSMs (**Supplemental Figure 2**). PSMs can be identified as individual peaks forming a typical pattern in reversed-phase HPLC chromatograms (**Supplemental Figure 2**) (22). We performed HPLC analysis to compare culture filtrates of the wild-type strain USA300 and the isogenic *eapΔH1ΔH2::ermB* mutant, either with or without addition of neutrophils and incubation for 24 hours. We found peaks representative of PSMα1, PSMα3, PSMα4 and for β-PSMs in the untreated culture filtrates of USA300 and the NSP inhibitor mutant (**Figure 5A**). Interestingly, neutrophil treatment of culture filtrates of USA300 led to reduction or loss of some of the typical PSMs peaks and the appearance of new ones, whereas incubation of the USA300*ΔeapΔH1ΔH2::ermB* mutant with neutrophils led to a pattern similar to that of the PSM-negative mutant (USA300*Δαβδ*) (**Supplemental Figure 2C**). The identity of the new peaks that exclusively appeared upon incubation of culture filtrates of USA300 with neutrophils but not in neutrophil treated USA300*ΔeapΔH1ΔH2::ermB* mutant was elucidated by mass spectrometry (MS). We found various truncated versions of PSMs in culture filtrates of USA300 and the NSP inhibitor mutant after 24 hours of incubation at 37°C (**Figure 5B** and **Supplemental Table 1**). In addition, the treatment of USA300 culture filtrates with neutrophils led to the appearance of further variants, which were hardly detected in untreated culture filtrates and in neutrophil treated culture filtrates of the USA300*ΔeapΔH1ΔH2::ermB* mutant (**Figures 5B–D** and **Supplemental Table 1**).

### PSM Degradation by NSP Leads to Reduced FPR2 Activation, Reduced Cytotoxicity as Well as Enhanced Susceptibility to Neutrophil Killing in the Absence of NSP Inhibitors

**Figure 6:**
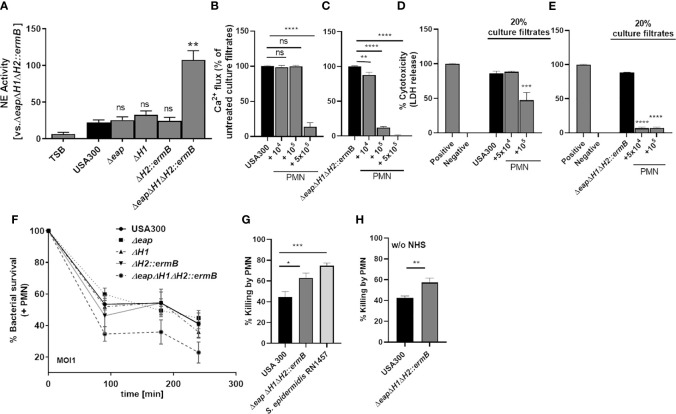
The combined deletion of the three NSP inhibitors enhances inactivation of PSMs by neutrophils in culture filtrates of USA300 and lead to enhanced killing of *S. aureus.* Incubation of neutrophils with bacterial culture filtrates of USA300 and isogenic triple-NSP inhibitor mutant induces release of NE. Inactivation of all three NSP inhibitors strongly enhances NE activity, whereas single knock outs do not **(A)**. Incubation of neutrophils for 24 h with USA300 or USA300*ΔeapΔH1ΔH2::ermB* leads to enhanced inactivation of FPR2 activity **(B, C)** and cytotoxicity **(D, E)** of the NSP inhibitor mutant compared to the wild type. The deletion of all three NSP inhibitors but not of a single NSP inhibitor impairs survival of *S. aureus* in the presence of neutrophils **(F)**. Killing of *S. aureus* USA300 compared to isogenic triple-NSP inhibitor mutant and *S. epidermidis* **(G)**. Data represent means ± SD of at least three independent experiments. ns, not significant, *P < 0.05; **P < 0.01; ***P < 0.001; ****P < 0.0001 significant difference *versus* USA300 **(A, G, H)** or untreated controls **(B–E)** as calculated by one-way ANOVA with Dunnett’s multiple comparisons test **(A–G)** or unpaired Students t-test **(H)**.

We then compared culture filtrates of the wild type and the various mutants for their capacity to induce NE release by neutrophils as assessed by measuring elastase activity (**Figure 6A**). Culture filtrates of all single mutants induced NE activity to the same extent as the wild-type culture filtrate. However, the activity of NE in the presence of the culture filtrate of the triple mutant was significantly higher. Furthermore, incubation of culture filtrates of the triple NSP inhibitor mutant with neutrophils for 24 hours led to a stronger inhibition of PSM-mediated FPR2 activation in HL60-FPR2 cells (**Figures 6B, C**) as well as cytotoxicity toward neutrophils (**Figures 6D, E**) compared to culture filtrates of the wild type.

Since PSMs strongly support the survival of *S. aureus* in neutrophils (27) by destroying the phagosome membrane, we assessed if enhanced degradation of PSMs by NSPs improved neutrophil killing of *S. aureus*. Indeed, simultaneous deletion of all three NSP inhibitors enhanced killing of *S. aureus* by neutrophils significantly (**Figures 6F, G**). Interestingly *S. epidermidis*, which lacks homologs of *S*. *aureus eap*, *eapH1* and *eapH2*, was better killed by neutrophils than wild-type USA300, probably because its PSMs were degraded and could not prevent neutrophil killing. *S. epidermidis* (**Figure 6G**). was still better killed than the triple NSP inhibitor mutant of *S. aureus* (**Figures 6 G, H**), which is probably due to the activity of additional *S. aureus* virulence factors produced by the triple NSP inhibitor mutant. Taken together these findings indicate that the NSP inhibitors are essential for the capacity of PSMs to promote *S. aureus* survival in neutrophils.

### NSP Inhibitors of *S. aureus* Are Necessary for FPR2 Dependent Bacterial Killing *In Vivo*

**Figure 7:**
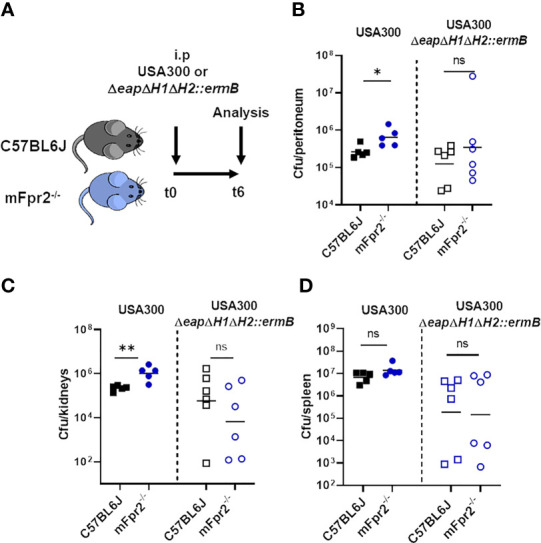
Absence of mFPR2 leads to impaired killing of wild-type USA300, but not of the NSP-inhibitor mutant *in vivo.* WT and isogenic mFpr2^-/-^ mice were intra-peritoneal infected either with USA300 or the isogenic NSP inhibitor mutant for six hours **(A)** mFpr2^-/-^ mice show enhanced bacterial numbers in the peritoneum **(B)** and in the kidneys compared to WT mice after i.p. infection with USA300 for six hours, but no difference between WT and mFpr2^-/-^ mice after infection with the USA300*ΔeapΔH1ΔH2::ermB* mutant **(C)**. Infection with the USA300*ΔeapΔH1ΔH2::ermB* mutant led to less bacterial dissemination into the spleen, but to no difference in bacterial load between WT and isogenic Fpr2^-/-^ mice **(D)**. Data in all panels represent geometric means from two independent experiments. Ns, not significant; *P < 0.05; **P < 0.01 significant difference *versus* the indicated WT mice infected with the USA300 wild-type (left) or with the isogenic USA300*ΔeapΔH1ΔH2::ermB* mutant as calculated by Mann-Whitney-U test.

It has been shown that an NSP inhibitor mutant of *S. aureus* Newman is less virulent in a murine intravenous infection model (18). To analyze the influence of NSP-mediated PSM degradation during infection, we performed infection experiments in mice carrying or not the mFpr2 receptor, which is necessary for the recognition of PSMs and activation of neutrophils. To this end, we infected wild-type (WT) and mFpr2^-/-^ mice intraperitoneally either with USA300 or with the corresponding NSP inhibitor mutant *ΔeapΔH1ΔH2::ermB* (**Figure 7A**). We analyzed leukocyte infiltration into the peritoneum (**Figure 7B**) and bacterial numbers in different organs (**Figures 7C, D** and **Supplemental Figure 3A**) six hours after infection. We observed that the number of infiltrated neutrophils and macrophages in the peritoneum was not significantly different after six hours of infection with USA300 or USA300*ΔeapΔH1ΔH2::ermB* as well as WT and mFpr2^-/-^ mice (**Supplemental Figure 3B, C**). However, infection with wild-type USA300 led to an enhanced bacterial load in the peritoneum and kidney of mice lacking mFpr2 compared to the wild-type mice (**Figures 7B, C**). The enhanced bacterial loads in the peritoneum and kidney of mFpr2^-/-^ mice might reflect either the enhanced phagocytosis *via* FPR2 activation or the enhanced early recruitment of neutrophils and macrophages in WT mice as described previously (25, 33). Notably, this difference was not observed in mice infected with the NSP inhibitor mutant (**Figures 7B, C**). Due to the lack of the NSP inhibitors in this strain, PSMs can be degraded by NSPs, which decreases neutrophil activation *via* FPR2. However, the numbers of bacteria of the mutant strain were equal in the peritoneum and kidney (**Figures 7B, C**) or reduced (**Figure 7D**) in the spleen compared to those of the wild-type *S. aureus*, indicating that *S. aureus* depends on the cytolytic PSMs while the neutrophil-activating FPR2-mediated PSM activity is less significant in this type of infection.

## Discussion

NSPs play important roles during bacterial infections, since they can eliminate bacteria or inactivate their virulence factors. Besides its proteolytic activity, the cationic enzyme NE has been shown to augment bacterial killing *via* its electrostatic interactions with the negatively charged bacterial membrane (34). Since *S. aureus* is able to modify its surface net charge through the activity of MprF and Dlt (35, 36), it is probably resistant to this effect. The idea that NSPs can inactivate toxins has been described for critical virulence factors of the Gram-negative bacteria *Shigella flexneri*, a gastrointestinal pathogen (8) and *Actinobacillus actinomycetemcomitans*, involved in the pathogenesis of periodontal disease (37). It has remained unclear, however, whether NSPs also degrade toxins of Gram-positive pathogens such as the PSM toxins of staphylococci and whether the inhibition of NSPs by NSP inhibitors might influence the efficacy of killing of *S. aureus* by neutrophils.

**Figure 8:**
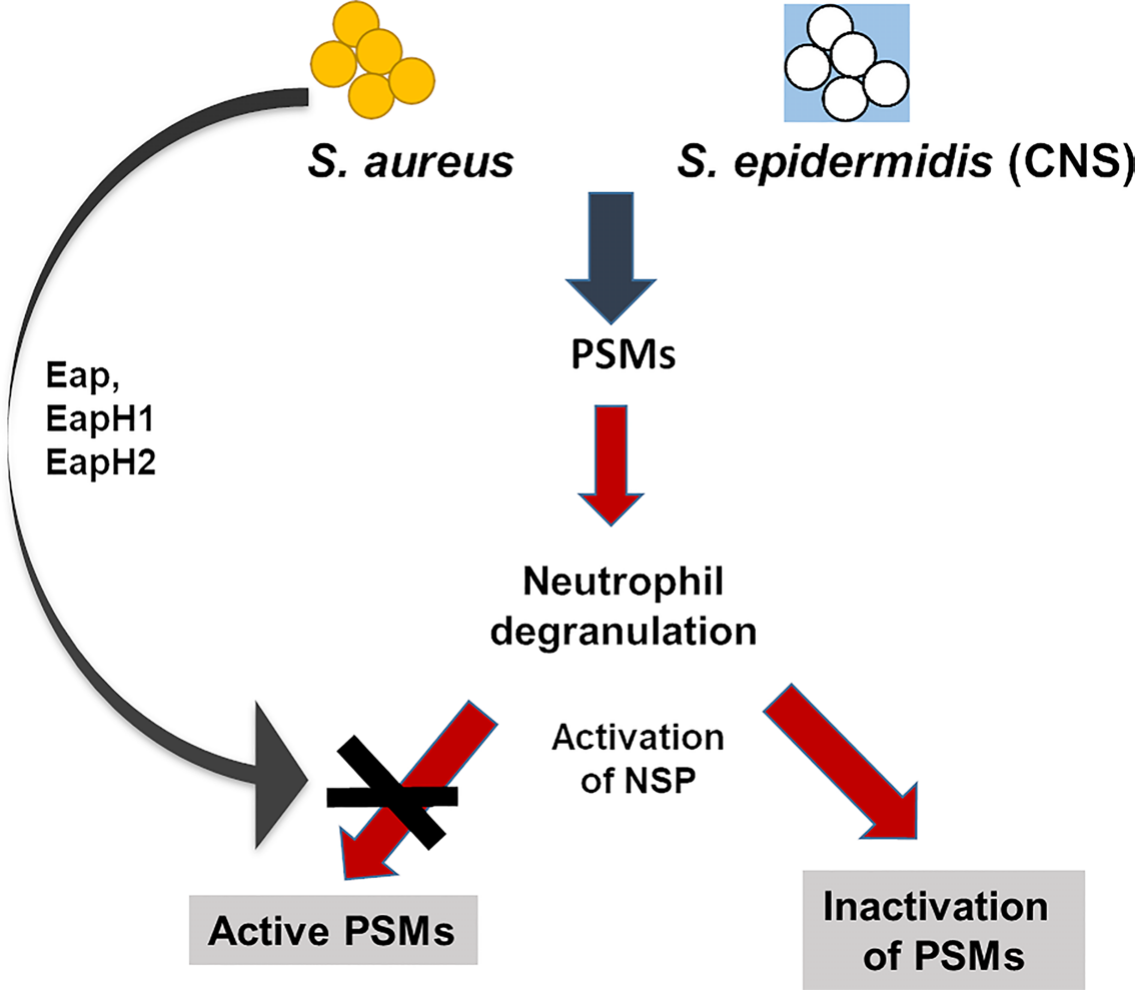
Influence of NSPs on PSMs in *S. aureus* and *S. epidermidis.* PSMs of *S. aureus* and coagulase-negative *S. epidermidis* activate neutrophils, induce degranulation and release of the NSPs NE, CG and PR3. Active NSPs degrade PSMs of staphylococci. *S. aureus*, but not the coagulase-negative *S. epidermidis* secretes three NSP inhibitors, the extracellular adherence proteins (Eap, EapHI, EapHII), which specifically inhibit NSPs. These NSP inhibitors prevent degradation of PSMs and thereby enhance the capability of *S. aureus* to survive better than *S. epidermidis* in the presence of neutrophils.

We demonstrate that NSPs are also very effective in neutralizing PSMs, which belong to the most potent *S. aureus* and *S. epidermidis* virulence factors, thereby underscoring the role of NSPs in the defense against a broad variety of bacterial toxins (**Figure 8**). While NSPs have no direct antibacterial activity against *S. aureus* they have been found to promote the killing of *S. aureus* for previously unknown reasons (18). Our study suggests that the degradation of PSMs by NSPs is responsible for the killing-promoting activity of NSPs and that *S. aureus* but not *S. epidermidis* is able to preserve PSM integrity by the release of its potent NSP inhibitors.

Although we found that PSMs can be degraded by NSPs, we observed that culture filtrates of USA300 are still able to activate FPR2 after incubation with relatively high amounts of neutrophils. Since *S. aureus* secretes NSP inhibitors, we rightly speculated that the secretion of these inhibitors could prevent full inactivation of PSMs in culture filtrates of *S. aureus*. In contrast, incubation of neutrophils with culture filtrates of coagulase-negative *S. epidermidis*, which produces PSMs but lacks NSP inhibitors, completely abrogated FPR2 activity. PSM production starts in the phagosome soon after phagocytosis, probably due to the local high bacterial density, which leads to activation of the quorum-sensing system Agr. It has been reported that alpha PSMs are responsible for the escape of *S. aureus* from the phagosome of neutrophils into the cytosol and for complete disruption of neutrophils (27) In case of macrophages it has been shown that the regulatory systems Sae and Agr are required in addition to the α- PSMs to obtain an full escape phenotype (28, 29) which means escape of bacteria from the phagosome and the cytosol into the extracellular space. Since serum lipoproteins prevent PSM activity, the intracellular role of PSM production in lysis and activation of neutrophils may be more important than the extracellular one. Eap is positively regulated by Sae, which gets activated for instance by α-defensins (38, 39), hydrogen peroxide, and other compounds from azurophilic granules (40, 41). Therefore, *eap* expression could prevent intracellular PSM degradation. However, we observed improved killing of *S. aureus* only when all NSP inhibitors are inactivated. Since these inhibitors are differentially regulated, also their extracellular release at later time points could be possible. FPR2 is expressed on the surface of host cells (42). Therefore, the observed FPR2-mediated effects in mice probably depend on PSMs in the extracellular space.

Coagulase-negative staphylococci also secrete alpha PSMs (22), but cannot escape from the phagosome, presumably because their PSMs are rapidly degraded and because they are unable to inactivate NSPs.

Release of the NSP inhibitor SmKI-1 by *Schistosoma mansoini*, a helminth pathogen responsible for gut bilharziosis, prevents specific neutrophil recruitment into the pleural cavity in response to carrageen injection (43). This observation underscores the neutrophil-specific anti-inflammatory potential of NSP inhibitors. Moreover, lack of alpha-1-antitrypsin, an endogenous NSP inhibitor, in patients with AATD (dysfunctional SERPINA1 gene, primarily found in the respiratory system) is characterized by severe respiratory problems caused by excessive degradation of lung parenchyma, increased inflammation and increased susceptibility to infections (44). On the one hand, the release of NSP inhibitors by *S. aureus* prevents degradation of PSMs and other virulence factors, which could exacerbate inflammation. On the other hand, inhibition of NSP could prevent NSP-induced endogenous inflammation, since NSP have a broad cleavage profile and also cleave various endogenous molecules, thereby inducing inflammation (45). Therefore, the release of three NSP inhibitors by *S. aureus* could be a hint that enhanced survival of *S. aureus* in contact with neutrophils is also associated with a balanced or even attenuated inflammation.

Our data indicate that better killing of coagulase-negative staphylococci by neutrophils compared to *S. aureus* depends at least in part on the lack of NSP inhibitors and the efficient degradation of PSMs by NSPs. Deletion of the three NSP inhibitors enhanced killing of *S. aureus* by neutrophils and led indeed to better killing of *S. aureus.* Nevertheless, individual deletion of the three serine protease inhibitors altered neither the activity of NE nor neutrophil killing of the *S. aureus* mutant compared to the wild type. These results are in line with recently published data indicating that the loss of one Eap protein can be compensated by the two remaining Eap proteins (13). *S. aureus* is known for the production of several virulence factors with redundant activity such as the different matrix protein adhesins, PSM peptides, or two-component pore-forming toxins. This redundancy is obviously also reflected in the expression of three NSP inhibitor genes by *S. aureus*. It has been shown that truncated PSM versions retain the ability to activate FPR2, in some cases even more than the original peptides (46–48). Neutrophil-treated culture filtrates of USA300 wild type did not only lead to decreased neutrophil degradation of PSMs compared to the triple-NSP inhibitor mutant, but also to the appearance of specific truncated PSM variants. This observation could imply that these specific PSM variants are responsible for the observed remaining FPR2-stimulating activity of the neutrophil-treated culture filtrates of the wild-type strain (48). However, this hypothesis requires further investigations with synthetic versions of these specific PSM variants. We also observed various truncated PSMs in culture filtrates of the wild type and the NSP inhibitor mutant after 24 hours of incubation, even in the absence of NSPs, probably resulting from the activity of secreted *S. aureus* proteases (49).

Although it has been shown that PSMs induce recruitment of neutrophils *via* FPR2 after three hours (33), mFpr2^-/-^ mice seem to compensate for the absence of mFPR2 to some extent after prolonged inflammation (50)., e.g. by enhanced expression of chemokine receptors (51). Such a compensatory effect could explain the comparable recruitment of neutrophils and macrophages into the peritoneum after six hours of infection in WT and mFpr2^-/-^ mice following i.p. infection with wild-type *S. aureus* or NPS inhibitor mutant. It has been shown that FPR2 activation through PSMs leads to enhanced phagocytosis and killing of *S. aureus* (25). Therefore, the enhanced bacterial load in the peritoneum and kidney of mFpr2^-/-^ mice after infection with USA300 is probably due to the lack of mFpr2. Since the lack of NSP inhibitors leads to inactivation of the PSMs by neutrophils we expected no difference between WT and mFpr2^-/-^ mice. Our *in vivo* data indicate that Eap protection of extracellular PSM allows for increased recognition of PSMs by FPR2 and the induction of FPR2-mediated neutrophil activation, leading to lower *S. aureus* CFUs. This seems to be detrimental for the bacteria. However, infection with the NSP inhibitor mutant led to an allover lower abundance of bacteria in the peritoneum and the organs, especially in the spleen compared to infection with wild-type USA300. Therefore, it is tempting to speculate that the overall advantages of PSM-mediated neutrophil destruction outweigh a possible loss of bacteria as a consequence of FPR2 activation and thereby enhanced bacterial killing by remaining neutrophils. More importantly, many *S. aureus* strains produce either FLIPr or FLIPr-like, which are inhibitors of FPR2 (52, 53). Since these inhibitors are specific for human FPR2, we cannot observe the effects of these molecules in mice. In addition, it has been shown that these inhibitors can also be degraded by NSPs. Inhibition of these NSPs by staphylococcal NSP inhibitors prevents at least in humans not only PSM cleavage, but also FLIPr inactivation and, as a consequence, FPR2 activation (13). These findings indicate that the NSP inhibitor mutant of *S. aureus* is less virulent than the parental strain, as described before (18).

We propose that by inhibiting NSPs, Eap, EapH1 and EapH2 protect PSMs from degradation and, thereby, represent an immune evasion mechanism of *S. aureus* that prevents neutrophil killing. This in part explains the more efficient killing of coagulase-negative staphylococci by neutrophils when compared to killing of *S. aureus*.

## Data Availability Statement

The original contributions presented in the study are included in the article/**Supplementary Material**. Further inquiries can be directed to the corresponding author.

## Ethics Statement

The institutional review board (IRB) of the University of Tübingen approved the study and all adult subjects donating blood provided informed consent. This study was done in accordance with the ethics committee of the medical faculty of the University of Tübingen that approved the study, Approval number 015/2014 BO2. The patients/participants provided their written informed consent to participate in this study. Experimental procedures involving mice were carried out according to protocols approved by the Animal Ethics Committees of the Regierungspräsidium Tübingen (IMIT1/18). Written informed consent was obtained from the owners for the participation of their animals in this study.

## Author Contributions

DK, RB, and CG designed and performed most of the *in vitro* experiments. DK and AP wrote the manuscript. DK, RB, and CG performed experiments. ML, KS, and DK performed animal experiments. MN and MS performed HPLC and LC/MS analysis. DS and SR provided recombinant Eap, EapH1 and EapH2 and helped with the knockout of Eap, EapH1 and EapH2. All authors contributed to the article and approved the submitted version.

## Funding

This study was funded by grants from the German Research Foundation with the funding grant numbers TRR156/2 – 246807620 to DK, AP, and DFG KR4395/3-1 to DK and the German Center for Infection Research (DZIF) to AP and DK. The authors acknowledge infrastructural support by the Cluster of Excellence EXC2124 Controlling Microbes to fight Infections, project ID 390838134.

## Conflict of Interest

The authors declare that the research was conducted in the absence of any commercial or financial relationships that could be construed as a potential conflict of interest.

## Publisher’s Note

All claims expressed in this article are solely those of the authors and do not necessarily represent those of their affiliated organizations, or those of the publisher, the editors and the reviewers. Any product that may be evaluated in this article, or claim that may be made by its manufacturer, is not guaranteed or endorsed by the publisher.
