# Supplementary material for: Staphylococcus aureus Depends on Eap Proteins for Preventing Degradation of Its Phenol-Soluble Modulin Toxins by Neutrophil Serine Proteases
Source: Front Immunol. 2021 Sep 6;12:701093. doi: 10.3389/fimmu.2021.701093 (PMC8451722; doi:10.3389/fimmu.2021.701093)
Supplement: Supplementary file 1 [file DataSheet_1.docx]

Supplementary Material

**Supplementary Table 1.**

| **Name** | **5‘-** | **Primer sequence** | **-3‘** | **Purpose** | **Length (bp)** |
| --- | --- | --- | --- | --- | --- |
| EapH1_F1_up | 5'- | CAACGAATTCTTTAACATGCAGTGTTATCCC | -3' | amplification and sequencing of flanking regions of EapH1 | 32 |
| EapH1_F1_down | 5'- | GATATTACACTAGATCTATAACACGTTTC | -3' | amplification and sequencing of flanking regions of EapH1 | 29 |
| EapH1_F2_up | 5'- | TGAAAATAGATCTATAGGGCAAGCGCTGAA | -3' | amplification and sequencing of flanking regions of EapH1 | 30 |
| EapH1_F2_down | 5'- | GGTATCGGTCGACTAACAGGTTCAAACGG | -3' | amplification and sequencing of flanking regions of EapH1 | 29 |
| 0883_5‘_fwd | 5'- | AAAGCAGATTTATCAAGAACAAAGGGC | -3' | amplification and sequencing of *ermB* insertion into EapH2 (5’ end) | 27 |
| 0883_3‘_rev | 5'- | CAATGACCTCTAACCCATCA | -3' | amplification and sequencing of *ermB* insertion into EapH2  (3’ end) | 20 |


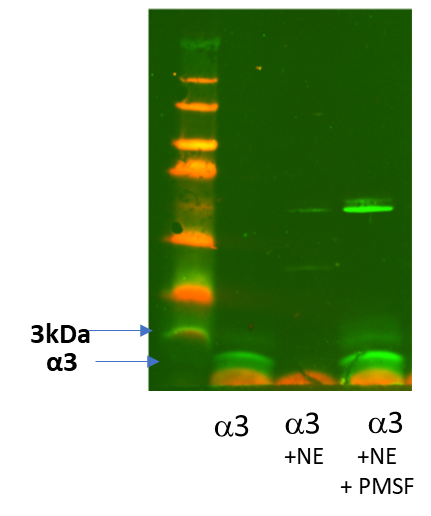


**Supplementary Figure 1.** **Inhibition of NSP by PMSF prevents degradation of PSMα3.** Digestion of PSMα3 with neutrophil elastase (NE) for one hour at 37°C with or without addition of 100µM PMSF. After centrifugation supernatant was used for Western Blot analysis

**
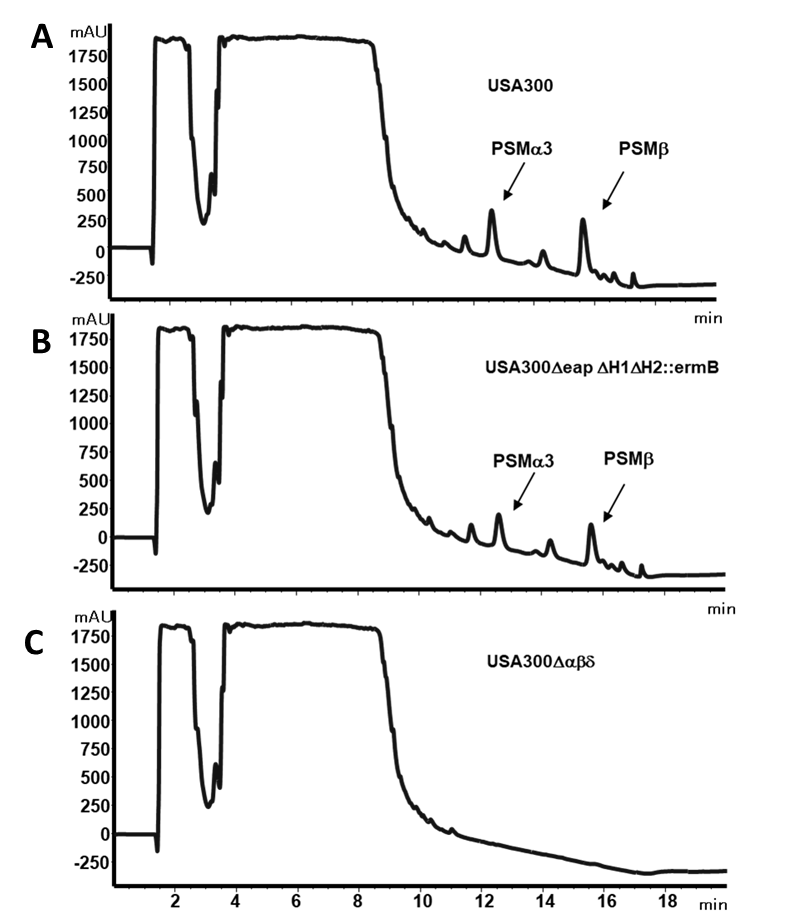
**

**Supplementary Figure 2.** **Amount of PSMs in culture filtrates of *S. aureus.*** HPLC results of 17 hours overnight cultures of USA300lac, of USA300*ΔeapΔH1ΔH2::ermB* and isogenic PSM deletion mutant USA300*Δαβδ*. Synthesized *S. aureus* PSMs are used as standards. USA300 wt and USA300*ΔeapΔH1ΔH2::ermB* show equal retention times (12,5 and 15,6 minutes for PSMα3 and PSMβ). Respective peaks lack in the PSM mutant USA300*Δαβδ.*

**
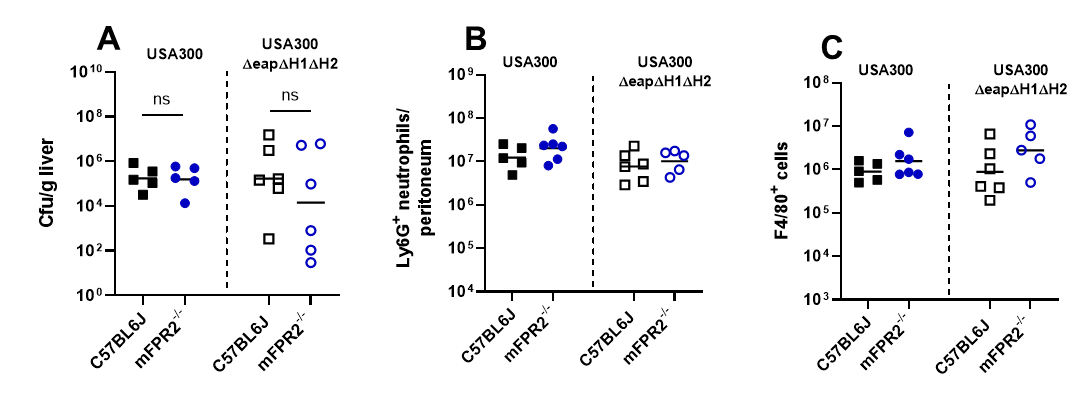
**

**Supplementary Figure 3.** Lack of NSP inhibitors does not lead to any difference between the bacterial load of WT and mFpr2^-/-^ mice. Data in all panels represent geometric means from two independent experiments. ns, not significant; versus the indicated WT mice infected with the USA300 WT or with the isogenic USA300*ΔeapΔH1ΔH2::ermB* mutant as calculated by Mann- Whitney-U test.
